# Resitting a high-stakes postgraduate medical examination on multiple occasions: nonlinear multilevel modelling of performance in the MRCP(UK) examinations

**DOI:** 10.1186/1741-7015-10-60

**Published:** 2012-06-14

**Authors:** IC McManus, Katarzyna Ludka

**Affiliations:** 1 Academic Centre for Medical Education, Division of Medical Education, University College London, Gower Street, London, WC1E 6BT, UK; 2 Division of Psychology and Language Sciences, University College London, Gower Street, London, WC1E 6BT, UK

## Abstract

**Background:**

Failure rates in postgraduate examinations are often high and many candidates therefore retake examinations on several or even many times. Little, however, is known about how candidates perform across those multiple attempts. A key theoretical question to be resolved is whether candidates pass at a resit because they have got better, having acquired more knowledge or skills, or whether they have got lucky, chance helping them to get over the pass mark. In the UK, the issue of resits has become of particular interest since the General Medical Council issued a consultation and is considering limiting the number of attempts candidates may make at examinations.

**Methods:**

Since 1999 the examination for Membership of the Royal Colleges of Physicians of the United Kingdom (MRCP(UK)) has imposed no limit on the number of attempts candidates can make at its Part 1, Part2 or PACES (Clinical) examination. The present study examined the performance of candidates on the examinations from 2002/2003 to 2010, during which time the examination structure has been stable. Data were available for 70,856 attempts at Part 1 by 39,335 candidates, 37,654 attempts at Part 2 by 23,637 candidates and 40,303 attempts at PACES by 21,270 candidates, with the maximum number of attempts being 26, 21 and 14, respectively. The results were analyzed using multilevel modelling, fitting negative exponential growth curves to individual candidate performance.

**Results:**

The number of candidates taking the assessment falls exponentially at each attempt. Performance improves across attempts, with evidence in the Part 1 examination that candidates are still improving up to the tenth attempt, with a similar improvement up to the fourth attempt in Part 2 and the sixth attempt at PACES. Random effects modelling shows that candidates begin at a starting level, with performance increasing by a smaller amount at each attempt, with evidence of a maximum, asymptotic level for candidates, and candidates showing variation in starting level, rate of improvement and maximum level. Modelling longitudinal performance across the three diets (sittings) shows that the starting level at Part 1 predicts starting level at both Part 2 and PACES, and the rate of improvement at Part 1 also predicts the starting level at Part 2 and PACES.

**Conclusion:**

Candidates continue to show evidence of true improvement in performance up to at least the tenth attempt at MRCP(UK) Part 1, although there are individual differences in the starting level, the rate of improvement and the maximum level that can be achieved. Such findings provide little support for arguments that candidates should only be allowed a fixed number of attempts at an examination. However, unlimited numbers of attempts are also difficult to justify because of the inevitable and ever increasing role that luck must play with increasing numbers of resits, so that the issue of multiple attempts might be better addressed by tackling the difficult question of how a pass mark should increase with each attempt at an exam.

## Introduction

When candidates take a high-stakes examination, some will fail. In most situations they are then allowed to resit the examination at a later date and sometimes they may retake the examination many times. Although a normal and accepted part of the examination system, retake examinations raise many questions, educational, statistical, moral and legal, few of which have been properly addressed in the literature.

In the UK the question of how resit examinations should be addressed has been focused by a consultation carried out by the General Medical Council (GMC), which asked among other things, 'whether there should be a maximum number of attempts, and if so, whether six attempts would be appropriate'. The minutes of the GMC Postgraduate Board of 20 April 2011 [1], reported that there were 104 responses, 77 from individuals and 27 from organizations. On the specific issue of the number of attempts, the only clear conclusion was that 'Opinion was divided', with the Academy of Medical Royal Colleges in particular emphasising that 'international consensus is not to limit attempts' (para 83). Although no clear conclusion was reached, the GMC document also stated that,

'The GMC's purpose is to protect patients. We continue to believe that the current situation... (in which) some specialties allow unlimited attempts to pass important, summative examinations, does not provide an acceptable basis for us to be sure that patients are being adequately protected. For so long as that assurance is missing, we believe that there is a legitimate role for the regulator in setting a backstop in these areas. However, any limits imposed by the regulator need to be based on evidence and command the maximum possible confidence and support of key interests.' (para 86).

The document continued, though, in the next paragraph,

Those conditions are not met at this time. It would be unfair to trainees to make decisions that limit the flexibility currently available to them without a very clear rationale and a broad measure of consensus.' (para 87).

There is little published information on how candidates perform when they repeat an examination on several or many occasions. Indeed, Ricketts [2], in a review stated, 'Following a literature search it became clear that there is no 'theory of resits'. There is much common practice but no evidence base for the interpretation of resit results' (p.351). Ricketts himself found only two relevant papers [3,4], to which we would add three others [5-7]. The paper by Pell *et al. *[4] asked whether standards in undergraduate resit assessments are comparable to those in main assessments and the Raymond and Luciw-Dubas paper [5], using a more limited version of the McManus model from 1992 [3], asks about the pass rate in candidates resitting a postgraduate examination. Raymond *et al. *[6] reported that there were large differences in the internal factor structure of marks from candidates passing or failing a clinical assessment at their first attempt but at a second attempt when they passed, the candidates had a factor structure similar to those passing at their first attempt. An additional paper from the same group has also demonstrated that measurement error is equivalent at first and second attempts [8]. Taken together, those findings do not constitute what the GMC referred to as a 'very clear rationale' on which policy might be implemented.

One of us, in a 1992 paper [3] which was one of Ricketts' two papers, suggested that a key educational and statistical issue concerning candidates passing a resit examination was, Did the candidates get better or did they just get luckier? Luck inevitably plays a role in any examination, particularly for candidates close to the pass mark. If a candidate happens to have been asked about topics on which they are ill-prepared then they may do less well than if given a selection of questions for which they had been better prepared. Regression to the mean is a universal phenomenon, and on that basis alone, candidates below the pass mark will, on average be less unlucky on the second than the first time, and therefore score more highly at a resit assessment (although that argument does assume that the mean mark is above the pass mark, which is not necessarily the case in very hard examinations). Regression to the mean, though, on its own, results in a poor educational justification for allowing resit examinations. The educational intent is that failing candidates will revise, will study more, and, therefore, will have acquired more knowledge or skills when retaking an examination, and on that basis will be more likely to pass at the resit than they were at the first attempt. The legal and moral arguments underpinning resits also require that the substantive knowledge and skills of candidates will genuinely have improved, for only then can the public be assured of the competence of practitioners. It is presumed that the public would not be reassured were mere luck to result in doctors now being qualified where previously they were not, even if there is hardly any member of the public who has not themself encountered the role of luck when taking examinations.

In statistical terms, the central theoretical issue for understanding resit examinations is to distinguish passing due to luck from passing due to an improvement in true ability, at least at the group level, even if it is not easy to determine that process at the individual level. With any process involving a chance component, random fluctuations will eventually result in a target being reached (and a useful analogy is with games in which one throws a die and has to get a six to start; when eventually a six is thrown it is not because the player got better but because they finally got lucky). Using the 1992 model presented by one of us [3], which of necessity could only use a limited amount of aggregated data, it was concluded that candidates on the MRCGP examination did indeed truly improve on their second and third attempt at the examination (first and second resits) but did not improve further at their fourth and fifth attempts. There was not however much statistical power to detect effects at those later attempts.

There is also a moral and legal argument, alluded to by the GMC, that if candidates do not (or cannot) truly improve at resits, then it would be reasonable that resits should not be allowed (or the public would not be protected as chance would continue to allow some candidates to pass despite their true ability level being below that required). A converse position, taking a candidate's perspective, is that if there is a true increase in ability across attempts then candidates should be allowed to continue sitting assessments until an appropriate amount of knowledge and skills is achieved, at which time the public can be seen as protected. A separate issue, which will be considered at the end of this introduction, and which does need proper consideration for the understanding of resit assessments, is whether the pass mark itself should be the same at resit examinations. This paper is not the place to go into such issues in detail and a more detailed analysis will be presented elsewhere. It should also be remembered that a scientific analysis of a topic such as resits can provide a better understanding of what candidates actually do on repeated attempts, but that while such a scientific analysis can inform policy, it cannot determine policy, which is subject to a range of other, specifically political, issues.

We know of no published reviews of the policy of different examination boards in relation to resits, but two recent unpublished reviews have considered the topic in relation to the GMC's consultation. One unpublished review considered UK postgraduate examinations (Khan, A. and Wakeford, R.: 'How many attempts should candidates be allowed at the CSA and the AKT?', unpublished manuscript) and reported no formal restrictions on numbers of attempts for examinations in MCEM, FCEM, MRCGP, MRCOG, MRCPCH, MRCP, FRCR, MRCS (Part A), DPM, MRCPsych, and MFOM (although in some cases training and employers imposed limitations) whereas there were restrictions on number of attempts for FRCA, DRCOG, MRCPath, MRCS (Acc&Emergency), MRCS (Part B), and FRCS (see the list of abbreviations for a detailed description of the various examination names). Unlimited attempts are also allowed at PLAB (Part 1) but PLAB (Part 2) is limited. An informal review of international requirements (Cochrane, K. 'Number of attempts allowed in international examinations', unpublished) found unlimited numbers of attempts at ECFMG in the US, and in postgraduate examinations in Canada, Australia and New Zealand, with limits on the number of attempts in South Africa. The situation for USMLE was more complex, with the examination itself allowing unlimited numbers of attempts (and 41 State Medical Boards set limits on attempts at one or all parts [9]). As Ricketts has said, 'It is easy to find out how different higher education institutions or certifying bodies treat resit examinations, but not why they are treated that way' (p.352), so that there are common practices and less common practices, but no evidential or theoretical bases for those practices.

The MRCP(UK) examination changed its policy on resits in the 1990s, deciding, for a number of reasons, that there would be no limit on the number of attempts which candidates were allowed on the three parts of the assessment, the change being introduced in the 1999/2 examinations. As a result the MRCP(UK) examination provides an excellent data set for assessing performance of candidates at unrestricted numbers of attempts over a long time period, with some candidates taking parts of the examination 20 times or more. MRCP(UK) is an international examination. That raises some issues in relation to the GMC consultation, because it is not clear to what extent the GMC consultation in the UK applies only to UK candidates taking the examination. Many candidates take MRCP(UK) outwith the UK, and never work in the UK; it might seem unreasonable therefore to apply GMC-led restrictions to them. However, attaining MRCP(UK) can be a means of gaining access to the UK Medical Register, at which point the number of attempts may become relevant to the UK medical authorities. Since the present study is mainly concerned with understanding the behaviour of candidates taking an examination on repeated attempts, and their origins or place of work are not relevant to that issue, differences between UK and non-UK candidates will be considered only briefly.

### A brief history of the MRCP(UK)

The Royal College of Physicians of London was founded in 1518, and its 'first duty... was to examine the credentials of persons claiming to have medical knowledge and to issue to them licenses to practise' [10]. With the passing of the Medical Act of 1858, the Licentiate became merely a qualification for general practice and a new Membership examination for physicians was instigated [11,12]. The first Membership examination was set by the London College in 1859, followed by the Royal Colleges of Physicians of Edinburgh and of Glasgow in 1861 and 1886. The three examinations merged as the MRCP(UK) in 1968. Each of the three parts of the examination currently has three diets ('sittings') per year, with 2010/3 indicating the third diet of 2010. The format of all three parts of the examination changed in the early 21^st ^century. Part 1 until 2002/2 used a multiple true-false (MTF) format, and it changed completely to a best-of-five examination (BOF) in 2003/2, after three hybrid diets from 2002/2 to 2003/1 with one BOF and one MTF paper. Part 2 changed to a BOF examination in 2002/2 and PACES (Practical Assessment of Clinical Examination Skills) replaced the old-style clinical examination in 2001 [13,14]. Some minor changes in the examinations have occurred since then, with the number of questions in Part 2 changing (details are provided elsewhere [15]).

Standard-setting for the examinations takes different forms. Each diet of the Part 1 and Part 2 examinations has a proportion of questions, typically about 30%, that have been used in a range of previous diets, and are reviewed by the Boards to check on content acceptability. Until the 2008/3 and 2010/1 diets of Parts 1 and 2, respectively, each question was reviewed by experienced examiners using an Angoff technique, which was then included in a Hofstee compromise method to set the pass mark, which could be assumed, as far as reasonable, to be broadly equivalent across different diets. For subsequent diets, statistical equating was used to set the pass mark, subject to review by the Boards. The questions which had been used previously were entered into an item bank and difficulties calculated using a one-parameter item-response theory model. Repeat (or marker or anchor) questions from previous diets could then be used to equate standards of current diets with previous diets and new items calibrated and entered into the bank to be re-used in future diets. PACES also changed in 2009/3 so that marks were skill-based rather than station-based [16]. For a transitional period for the three diets from 2009/3 to 2010/2 pass-fail decisions for PACES were based on the total score achieved and then from 2010/3 onwards each of the seven skills had a separate pass mark, with candidates having to pass in all seven skills in order to pass the assessment [16]. PACES is a clinical examination using real patients, who are inherently variable and, therefore, the setting of passmarks uses implicit criterion-referencing, case difficulty being calibrated by examiners before each examination, and judgments of individual candidates made against clear criteria for each individual skill. For further information on all three parts of the examination see http://www.mrcpuk.org/SiteCollectionDocuments/MRCP_Regulations.pdf.

The analyses in the present paper use some complex statistical techniques, in particular multilevel modelling, which have not been used much in medical education. As a result the analyses presented here are, to a certain extent, expository, in order that readers will be able to understand the way in which the techniques are used and the sort of questions that can be answered.

### 'True ability'

The term 'true ability' is used on various occasions throughout this paper and it is potentially very misleading if misconstrued. We use it entirely in a technical, psychometric, sense to refer to the underlying, latent ability possessed by an examination candidate, which in an actual examination combines with various random processes ('error', 'noise') to manifest as a mark representing performance [17]. The term 'true ability' specifically does not refer to the actual clinical ability of a doctor in their practice, which is a separate construct.

## Methods

Data were extracted from the MRCP(UK) database on 6 April 2011 and contained marks for 24 diets of Part 1 from 2003/2 to 2011/1, for 25 diets of Part 2 from 2002/3 to 2010/3 and for 29 diets of PACES from 2001/1 to 2010/3 (Note: there were only two diets of PACES in 2001).

### Rescaling of marks

As described earlier in the 'brief history', marking schemes have changed. For convenience marks for Part 1 and Part 2 are rescaled to the percentage correct marks applying in the diets of 2008/2 and 2009/3, respectively, the base forms for subsequent statistical equating. These marks in principle are in the range 0% to 100%, although there is no negative marking and pure guessing would result in a score of 20%. Pass marks differed at each diet (as a result of differing question difficulties) and, therefore, all marks here are expressed as percentage points relative to the pass mark, so that a score of zero is a minimal pass and all negative marks are fails. PACES marks until 2009/2 used a different marking scheme, with marks in the range 14 to 56 [14] and 41 being the pass mark on all occasions. Marks since 2009/3, which used skills-based marking [16], were rescaled to the old marking scheme and then all marks expressed as marks above or below 41, so that 0 is a pass and negative marks are fails. Note that there is no direct comparability between a mark of, for example, +5 on Part 1, Part 2 or PACES.

### Statistical analyses

Conventional statistical analyses were carried out using SPSS 13.0, multilevel modelling used MLwiN 2.16 and non-linear multilevel modelling used the NLMIXED procedure in SAS 9.2 [18]. Multilevel modelling [19,20] can be used to carry out latent variable growth curve modelling [21], which itself is closely related to structural equation modelling [22].

### Auto-regressive modelling

Longitudinal models can be of two types, which in the ARIMA (auto-regressive integrated moving average) specification for time series are described as Auto-Regressive (AR) and Moving-Average (MA). Moving average models assume that for any particular datapoint or series of datapoints there is a true or latent value and the actual value is dependent on the latent value coupled with random error, typically due to measurement error. An alternative approach is auto-regression, whereby the Nth measure depends not on a latent value, or even on the latent value at time N-1, but on the actual value at time N-1. Such models may be useful when considering examinations, not least as if a candidate has a true measure of 49, but measurement error means they are over the passmark of 50, achieving a mark of 51, subsequent behaviour in the real world depends on the actual value of 51 (the examination has been passed), rather than the latent value (the candidate has a true performance below the pass mark). Although in principle AR and MA approaches can be combined, MLwiN cannot fit AR components, and therefore they have not been included here. It is possible that auto-regressive components would form a useful area for future exploration and it is also possible that our model may have minor errors in it due to the omission of AR components.

### Data centering and the coding of time

A key technical issue in any multi-level modelling (MLM) [23], particularly when the data are longitudinal (growth-curve modelling) [24], involves the coding of time and the form of centering that is used (or not used). For MLMs in general, Enders and Tofighi [23] (p.122) quote Kreft *et al. *[25], who say, 'There is no statistically correct choice among RAS, CGM (centering using the global mean), and CWC (centering within clusters)' (p.17) and themselves conclude, 'The decision to use CGM or CWC cannot be based on statistical evidence, but depends heavily on one's substantive research questions' (pp.135-136). Enders and Tofighi's rules of thumb include whether the key interest is in the level 1 association of X and Y, in which case CWC is appropriate, or in a level 2 prediction, when GCM is appropriate. Longitudinal models (growth curve models) have additional considerations, and Biesanz *et al. *[24] follow Raudenbush [26], who 'repeatedly emphasized parameterizing growth curve models to address the specific substantive questions of import to the researcher' (p.31). In answer to their own question of 'How should time be coded?', Biesanz et al. answer that, 'time should be coded to produce parameter estimates that are more easily and readily interpretable, and 'time should be coded to focus attention and understanding where the primary substantive questions lie' (p.41). We therefore recode time so that the first attempt at an examination is at time zero and in our main analyses we do not use centering (that is, we use raw-dating modelling, RAM). However, a final subsection of the Results section compares the very different conclusions reached by RAM and CWC which we then interpret.

## Results

Data were available for 70,856 attempts at Part 1 by 39,335 candidates at 24 diets, for 37,654 attempts at Part 2 by 23,637 candidates at 25 diets, and for 40,303 attempts at PACES by 21,270 candidates at 29 diets. For the present analyses, all candidates have been included, many of whom are non-UK graduates, and who on average perform somewhat less well than UK graduates, although that makes no difference to the present analyses.

### Censoring and truncation

In interpreting these data it should be remembered that they are censored and truncated. The data are right-censored in that for recent diets some candidates may have taken the examination only once or twice and will continue in the future to make more attempts. The data are also left-censored in that at the first attempt for which these data were available, some candidates were already on a second or higher attempt (or may already have passed, for example, Part 1, and so results are only available for Part 2 or PACES). The data are also truncated in that some candidates voluntarily withdraw from the examination at, for instance, the nth attempt, without having passed. The data are also truncated in that if a candidate passes the examination on the nth attempt then necessarily no data are available for their (n+1)th and higher attempts.

### Number of attempts

**Figure 1:**
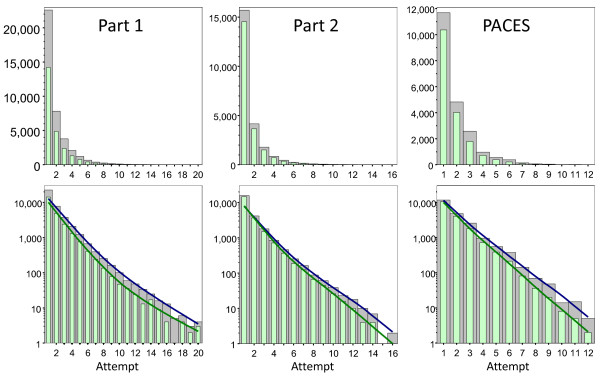
**Numbers of attempts at Part 1, Part 2 and PACES**. Top row: The figures show, for Part 1, Part 2 and PACES, the highest number of attempts at the examination (grey bars), and if the examination was passed, the attempt at which it was passed (pale green bars). Bottom row: The same data as in the top row but the ordinate is on a logarithmic scale. The fitted lines are lowess curves, blue for highest attempt and green for attempt at which the examination was passed. PACES, Practical Assessment of Clinical Examination Skills.

Figure 1 shows, for each part of the examination, the highest number of attempts recorded for each candidate and the attempt at which, if any, the examination was passed. Results are shown up to attempt 20 for Part 1, attempt 16 for Part 2, and attempt 12 for PACES. The highest number of attempts recorded for Part 1 was 26 (two candidates), for Part 2 was 21 (one candidate), and for PACES was 14 (one candidate). The top row shows that the distributions are heavily skewed to the left, so that it is difficult to see the right-hand end of the distribution. The lower row shows the same results plotted on a logarithmic ordinate. To a first approximation, except for the first few attempts, the distributions are exponential, falling away by a similar proportion at each attempt. The lines for the attempt at which an examination is passed are generally steeper than the line of the highest attempt, implying that at each attempt a smaller proportion of candidates passes.

As well as attempts at each individual part, the total number of attempts to pass all three parts of the examination was calculated (although this is not straightforward, as not all candidates passing Part 1 go on to take Part 2 and so on). Since the concern is mainly with those passing MRCP(UK) overall, the analysis is restricted to the 10,951 individuals in the database who had taken and passed all three parts of the examination. The minimum number of attempts to gain MRCP(UK) is, of course, three (one for each part), the mean number of attempts was 5.01 (SD 2.72), the median was 4, and 36.8% of candidates passed in three attempts, 21.7% took four, 13.9% took five, and 7.7% took six (an average of twice at each part). The 90^th ^percentile to pass all three parts was 8, the 99^th ^percentile was 15, and the maximum number of attempts to pass was 35. Although not shown here, the distribution was also exponential, being almost perfectly straight when plotted on a logarithmic ordinate.

### Candidates passing after the sixth attempt

Considering only the 10,951 candidates who passed all three parts of the examination, 1.8% (196) passed Part 1 after the sixth attempt, 0.5% (54) passed Part 2 after the sixth attempt, and 0.3% (152) passed PACES after the sixth attempt. Overall, 2.8% (308) passed one or more parts after the sixth attempt (and hence would fall foul of a limitation of six attempts), but only 0.30% (33) passed after more than 18 attempts in total (that is, an average of more than six attempts at each part).

### UK doctors

Although the main analyses will not, for reasons already discussed, be separating UK trained doctors from non-UK trained doctors, here we provide some brief descriptive statistics on three groups: UK graduates, UK trainees (identified probabilistically as non-UK graduates with a UK correspondence address), and non-UK doctors (neither UK graduates nor a UK correspondence address). For the 6,633 UK graduates, the mean total number of attempts to pass all three parts was 4.2 (SD 1.8), the median was four, and 47.1% of candidates passed in three attempts, 23.8% took four, 13.0% took five, and 6.1% took six (an average of twice at each part). The 90^th ^percentile was six, the 99^th ^percentile was 11, and the maximum number of attempts to pass was 30. A total of 0.8%, 0.1% and 0.05% of candidates passed Part 1, Part 2 and PACES on the 7^th ^or higher attempt and 0.9% passed at least one part on the 7^th ^or higher attempt. For the 2,411UK trainees, the mean total number of attempts to pass all three parts was seven (SD 3.6), the median was six and 12.4% of candidates passed in three attempts, 15.5% took four, 14.6% took five, and 11.1% took six (an average of twice at each part). The 90^th ^percentile was 12, the 99^th ^percentile was 18 and the maximum total number of attempts to pass was 35. In all, 4.7%, 1.5% and 2.7% of candidates passed Part 1, Part 2 and PACES on the seventh or higher attempt and 8.1% passed at least one part on the seventh or higher attempt. For the 1,907 non-UK doctors, the mean total number of attempts to pass all three parts was 5.2 (SD 2.7), the median was four, and 31.5% of candidates passed in three attempts, 22.1% took four, 15.9% took five, and 8.8% took six (an average of twice at each part). The 90^th ^percentile was nine, the 99^th ^percentile was 16, and the maximum number of attempts to pass was 24. In all, 1.6%, 0.6% and 0.8% of candidates passed Part 1, Part 2 and PACES on the seventh or higher attempt and 2.9% passed at least one part on the seventh or higher attempt.

### Non-multilevel analyses

Because the data being analyzed are necessarily multilevel, simple descriptive statistics which do not take that structure into account are potentially very misleading. However, since that is the immediate way in which most users will encounter such data, we explore the data for the Part 1 examination only to give a sense of how the data look and the problems of interpreting them.

**Figure 2:**
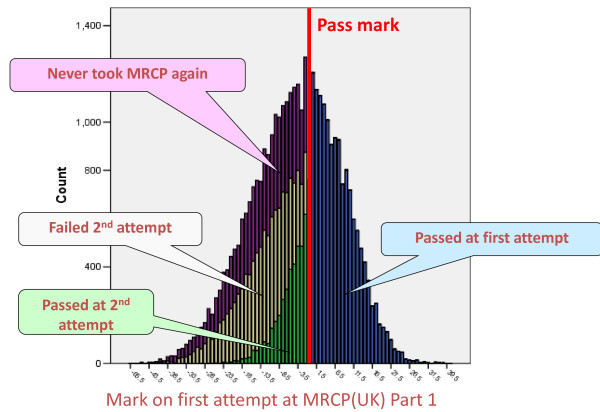
**Distribution of marks attained at the first attempt at MRCP(UK) Part 1, according to whether the examination was passed (blue), the examination was passed at the second attempt (green), the examination was failed at the second attempt (pale yellow), or the examination was not taken again (purple)**.

Figure 2 shows a histogram of the marks attained by all candidates on their first attempt at Part 1. The distribution is approximately normal, but skewed somewhat to the left, with a few candidates performing very badly. The marks in Figure 2 have been divided according to the outcome of candidates' second attempt at Part 1. Some of the candidates, shown in blue, do not take Part 1 again as they passed at their first attempt. The candidates in green and pale yellow took the examination a second time, those in green passing on the second attempt, whereas those in pale yellow failed on the second attempt (and they have rather lower marks at their first attempt than those who passed on the second attempt). There is also a large and rather problematic group, shown in purple, who never took Part 1 again (and in some cases that was despite having a mark only just below the pass mark, so they would have had a high chance of passing at a second attempt). Nothing further is known as to why the candidates in purple did not take the examination again, although it may be that some had taken the examination prior to a final decision about career choice and the examination had subsequently become irrelevant to their needs.

**Figure 3:**
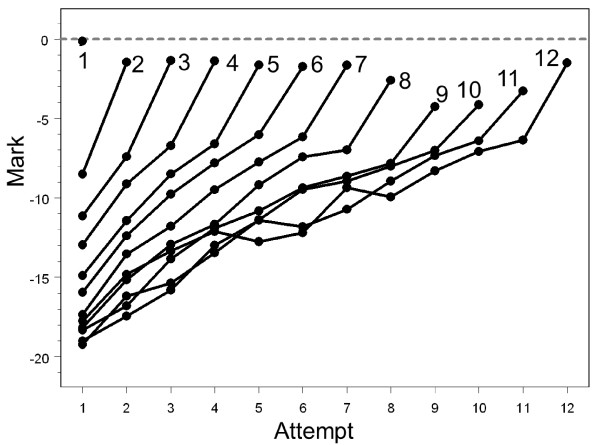
**The average mark at each attempt at the Part 1 examination according to the number of attempts made at the examination, from 1 to 12**. N varies from 19 to 22,602. The dashed grey line shows the pass mark.

**Figure 4:**
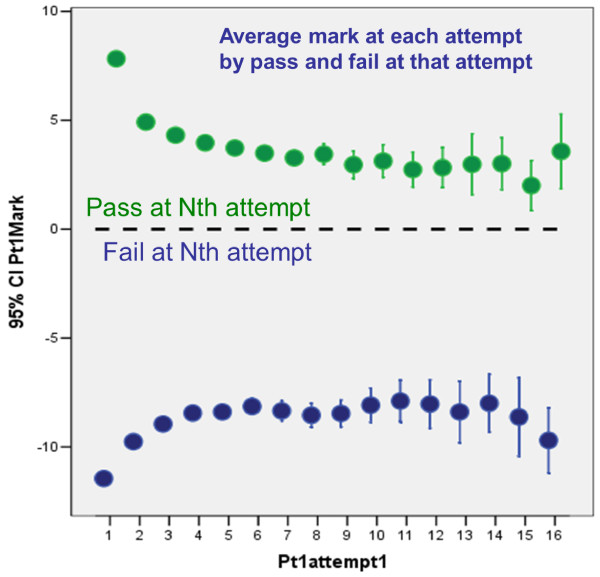
**Average mark of candidates at each attempt separately for those who pass or fail**.

Figure 3 shows the average marks of candidates at each attempt at the examination for those who had a total of one, two, three, up to twelve, attempts at the examination. The lines 'fan out', those taking the examination only two or three times having steeper slopes than those taking it ten or more times. Even for those taking the examination once, the mean mark is less than the pass mark (zero) and that is because, as in Figure 2, this group consists of a mixture of those passing the examination (with marks ≥ 0) and those failing the examination (marks < 0) but not going on to take it again. All groups, even those taking the examination up to twelve times, appear to be improving across all attempts. There is also clear evidence of a 'jump' at the last attempt which is due to some candidates exceeding the pass mark and therefore not needing to take the examination again. The groups at each attempt who pass or fail the examination are separated out in Figure 4, which shows the average mark of candidates on their nth attempt, according to whether they passed or failed at that attempt. Now, and not surprisingly, the average mark of those passing is > 0 and of those failing is < 0. More interestingly, those who pass at later attempts have lower marks when they eventually passed than those who passed at earlier attempts; and conversely, those who fail at later attempts have higher marks than those who fail at earlier attempts. Also of particular interest is that the lines seem to flatten out after about the seventh or so attempt.

**Figure 5:**
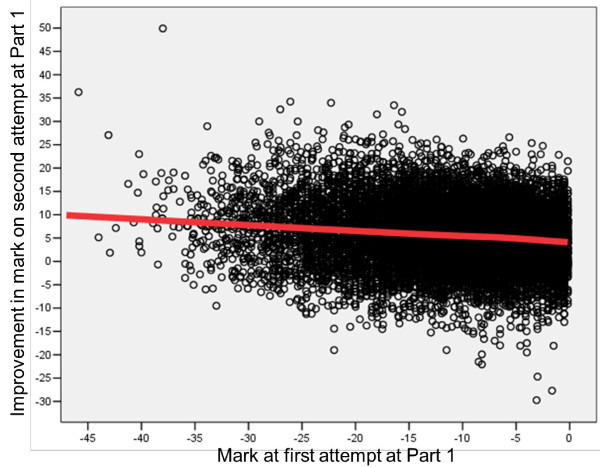
**Performance of candidates at the second attempt at MRCP(UK) Part 1 in relation to performance at the first attempt at Part 1**. Note that the fitted line is a Lowess curve, although it is almost indistinguishable from a straight line except for a slight change in direction between -5 and 0. MRCP(UK), Membership of the Royal Colleges of Physicians of the United Kingdom.

Figures 2, 3 and 4 do not show longitudinal results of individual candidates. In contrast, Figure 5 shows the marks of candidates at their second attempt, in relation to their performance at the first attempt (and, of course, all of these candidates had marks of less than zero at the first attempt because they had failed previously). On average, candidates do better on their second attempt than their first, with very poorly performing candidates improving the most. Although the latter is what might be expected from regression to the mean, it is worth noticing that the mean on the first attempt of all candidates is actually at about -4, and, therefore, it might be expected that those with marks greater than -4 would do worse on a second attempt, which they do not do.

### Multi-level modelling

Interpreting Figures 2, 3, 4 and 5 is possible, but is not straightforward, mainly because the data are inherently multi-level. A better approach is to model the data formally and for that MLMs are needed, with individual examination attempts being at level 1, and candidates being at level 2. Since MLMs can be complex, to prevent the flow of the argument being disrupted or becoming too confusing in the main text, details are presented in Additional File 1. Readers with a technical understanding of MLMs are referred to that file, whereas other readers should hopefully be able to understand the key ideas of the main paper without needing to refer to the details.

It should be pointed out that MLMs can model two very separate aspects of the data, and these will be considered separately. Firstly MLMs can look at fixed effects, which consider the average performance of all candidates, and secondly it can ask questions about random effects, which consider how candidates differ in their performance around a fixed effect. Fixed effects are mainly of interest for considering the overall process, whereas random effects are of much greater interest for understanding the educational and psychological processes which underpin the changes in performance of candidates retaking examinations.

### Model M1: group level (fixed effect) analyses

**Figure 6:**
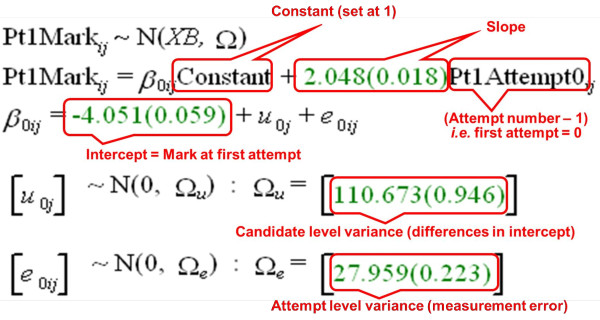
**Model M1 (see text)**. The model is fitted in MLwiN and shows the 'Equations' screen from MLwin (black and green fonts), annotated in red to indicate the meaning of the various components. MLwiN, Multilevel Modelling for Windows.

A simple MLM for the Part 1 data (model M1) is shown in Figure 6, as annotated output from MLwiN. Fitted parameters are shown by MLwiN in green and give the estimate followed in brackets by its standard error. At the measurement level (level 1) there is variability resulting from individual attempts by candidates, and this has a variance of 27.96. Individual attempts by candidates are nested within the second, candidate, level, the variance of which is 110.67, so that 79.8% of the total variance is at the candidate rather than the attempt level. The variances at the candidate and attempt levels are random factors. There are two fixed factors in model M1, both at the candidate level, and these are fitted as a conventional regression model according to the attempt number. For convenience, attempt at the examination is indicated by the variable Attempt0, which is the attempt number minus one, so that the first attempt is 0, the second attempt is 1, and so on. That has the useful feature that the 'intercept' or 'constant' of the regression model is performance at the first attempt at the examination, and the slope indicates the average improvement in performance between the nth and the n+1th attempt. The intercept is -4.051 and corresponds to the average mark of the candidates at their first attempt. The slope is 2.048 and it has a standard error of 0.018, meaning it is very significantly different from zero. On this model, candidates therefore show significant improvement at later attempts on the examination, improving on average by 2.048 marks at each resitting, with the assumption that the improvement is identical at all resittings.

### Model M2: non-linear modelling in MLwiN using dummy variables

**Figure 7:**
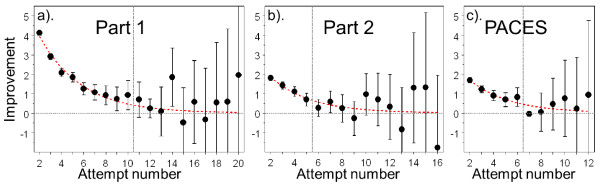
**Solid circles show the improvement in performance at each attempt at a) Part 1, b) Part 2 and c) PACES**. These are the estimates of the dummy variables in model M2, and show the estimated change in mark (± 2 SEs) from Attempt n-1 to Attempt n, with the abscissa showing n. The horizontal dashed grey line shows zero (no change) and the vertical dashed line shows the horizontal position beyond which the 2 SE confidence interval first includes zero. The dashed red line indicates the expected change for the negative exponential model fitted as model M3. PACES, Practical Assessment of Clinical Examination Skills.

Although model M1 is simple, it is clearly too simple as it implies that candidates improve by the same amount at each resit (and if that continued for ever then as the number of attempts increases the performance of each candidate would eventually reach the pass mark and all candidates eventually would pass the examination). A more intuitive approach is adopted in Model M2 in which candidates improve less and less at each attempt, perhaps eventually 'topping out' at some level. That possibility can be examined in MLwiN by fitting a purely empirical model in which there is a separate 'dummy variable' for each attempt, Dummy7, for instance, indicating by how much performance at the seventh and subsequent attempts is better than performance at the sixth attempt. The details of the fitting of model M2, are provided in Additional File 1. Here we restrict ourselves to showing, in Figure 7, the estimates of the dummy variables at each attempt, along with their confidence intervals. The left-hand graph shows the estimates for Part 1 and it can be seen that the extent of the improvement at each step falls with each attempt but that the improvement is still significant from the ninth to the tenth attempt. From the 11^th ^attempt onwards the confidence intervals for the improvements include zero and the curve is essentially flat. We can therefore conclude that, on average, candidates are showing a significant improvement at least until their tenth attempt. Figure 7 also shows equivalent analyses for Part 2 and PACES, where the improvement is significant until the fourth attempt and the sixth attempt, respectively.

### Model M3: fitting a negative exponential curve using SAS

Figure 7 suggests that candidates improve at each attempt at an examination but that the extent of the improvement becomes less with each attempt, eventually seeming to 'top out' (that is, the improvement at each attempt approaches zero). Model 3 adopts a natural way of modelling such performance which is derived from the psychological literature on motor-skill learning, as a negative exponential, of the form:

$$a=m-\left(m-s\right).e^{b\left(n-1\right)}$$

where the level of achievement, a, depends on the starting level of achievement (s), the maximum possible level of achievement (m), the attempt number (n; 1 indicates the first attempt), and a parameter (b), which normally will be negative, which determines how fast is the rate of change from the starting level to the maximum level. Because of the exponential function, the achievement only approaches the maximum level asymptotically, becoming ever closer in smaller and smaller steps, but never actually reaching it.

**Figure 8:**
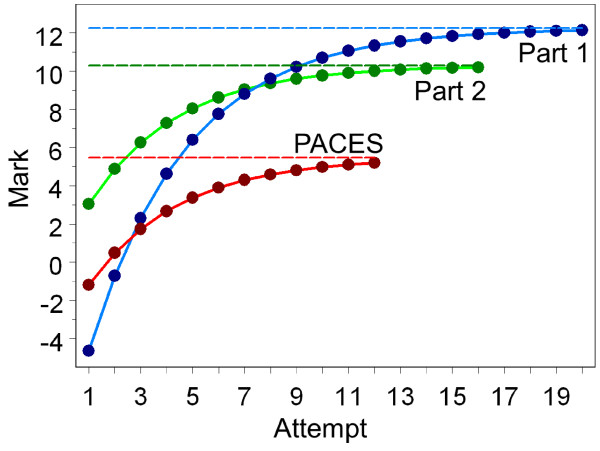
**Shows fitted negative exponential curves for Part 1 (blue), Part 2 (green) and PACES (red)**. The horizontal blue, green and red dashed lines are the estimated maximum (asymptotic) levels for Part1, Part2 and PACES. Note that the three examinations are not on equivalent scales and, therefore, no direct comparison between absolute levels should be made. PACES, Practical Assessment of Clinical Examination Skills.

Negative exponential models of this sort cannot be fitted in MLwiN, but can be fitted using the NLMIXED procedure in SAS [18]. Figure 8 shows the fitted curves for the Part 1, Part 2 and PACES examinations and it can be seen how the curves approximate to but do not reach the maximum levels. The dashed lines in Figure 7 also show the expected change at each attempt (in effect the first differential) and these correspond well to the effects found using the dummy variables method in MLwiN.

### Individual level (random effects) analyses

The fixed effect models described so far show that at the group level, the performance of candidates continues increasing, albeit at an ever-diminishing rate, over many attempts, and that for the Part 1 examination there is a significant improvement even between the ninth and tenth attempts at the examination. The results are well fitted by a negative exponential curve, implying that there is some maximum level of achievement towards which candidates are rising. However, such models do not take differences between candidates into account and, for instance, the implicit assumption is made that a candidate starting with a low mark at the first attempt would rise at the same rate and towards the same maximum as would a candidate starting at a high level on their first attempt. Whether or not that is the case has important implications for educational theory and for understanding the difference in performance of candidates. It can be modelled using random effects as well as fixed effects using MLMs.

Multilevel modelling was developed within an educational context in order to assess not only how groups of individuals behave on average, but also how individuals and members of groups behave in different ways [20]. In this paper we will firstly describe a simple (linear) random effects model for the Part 1 data, in order to give non-expert readers a flavour of what random effects models can do, and then we will go on to describe more complex random effects models using a dummy variables approach (using MLwiN) and the negative exponential curve (using *SAS*).

### Model 4: A linear random effects model using MLwiN

**Figure 9:**
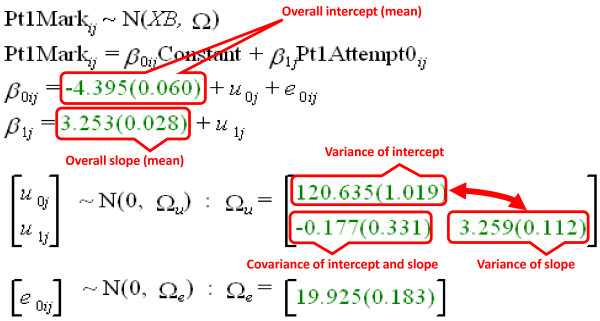
**Annotated output from MLwiN to show fitting of model M4, in which there is linear growth across occasions, and also random variation across candidates in the slope and intercept, and covariation between the slope and intercept**. Annotations on the output are shown in red. MLwiN, Multilevel Modelling for Windows.

Model M1 in Figure 6 shows a simple, fixed effects linear model of Part 1 performance, with only the variance (error terms) at each level being random. The important, interesting parts of the model are the intercept, which shows the level of performance of candidates on their first attempt at the examination and the slope, the amount by which, on average, performance increased at each attempt. However, it is not realistic to assume that every candidate starts at the same point at their first attempt and increases at the same rate at subsequent attempts. Model M4 in Figure 9 shows annotated output from MLwiN in which both the intercept and the slope are random variables, so that candidates can start at different levels, they can improve at different levels and, more subtly, there can be a co-variation (correlation) between the starting point and the rate of improvement of candidates. As before, attempt is modelled as Attempt0, where the first attempt is scored as zero, the second attempt as one, and so on. There are still fixed effects of the intercept and the slope, and these take values of -4.39 and 3.25, and these are broadly similar, if slightly different, to those found earlier in M1, and they represent the mean starting point and the mean slope. Model M4, however, also provides an estimate of the variance of the intercept, which is 120.63, so that the standard deviation is sqrt(120.63) = 10.98, meaning that 95% of the starting values are expected to be in the range -4.39 ± 1.96 × 10.98, that is -25.9 to +17.1. Clearly with such values, some candidates will pass at their first attempt and others will have very poor performances. Just as the intercept (starting value) can vary between candidates, so also can the slope, which has a variance of 3.259, corresponding to a standard deviation of sqrt(3.259) = 1.81, so that 95% of the slopes, the measures of improvement, will be expected to be in the range -.29 to +6.78. For the majority of candidates the slope will be positive and they will improve across attempts (presumably because of extra study) but a small minority seem to be getting worse (which might occur due perhaps to forgetting, or perhaps bad luck). A very important feature of M4 is that there is also a covariance of the intercept and the slope; a negative value would mean that as the starting point gets higher, so the rate of increase becomes smaller, whereas a positive value would mean that as the starting points becomes higher so the rate of increase is greater. Both effects would have their implications for understanding candidate behaviour. The actual co-variance is -.0177, which is equivalent to a correlation of -.0177/sqrt(120.63 × 3.259) = -.0089. In this case the co-variance (and hence also the correlation) is actually not significant as its standard error is .331, meaning that it is not significantly different from zero. For this simple model it seems that the rate of improvement of candidate performance is unrelated to the starting level. That seems a little unlikely, but the model fitted is linear, and as we saw with M1, that is also an unrealistic assumption, even if it is useful for simplifying the model fitting. The next step is, therefore, to consider non-linear models. Before doing that, though, it is important to notice that M4 is also an improvement over M1, because the variance due to measurement error, which was 27.96 in M1 has fallen to 19.93 in M4, so that more variance is now being accounted for by (systematic) differences between candidates rather than by random error due to testing (85.6% rather than 79.8%).

### Model 5: A random effects, negative exponential model using SAS

**Figure 10:**
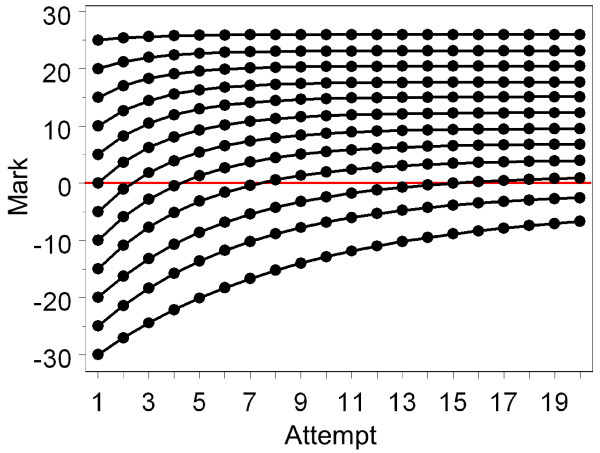
**Shows the expected behaviour at each attempt, based on the fitted negative exponential model, M3, of candidates whose mark at the first attempt at Part 1 varies from 30 marks below the pass mark to 25 marks above the pass mark**. Note that the maximum levels and the rate of increase co-vary both with the starting level and with each other. The red horizontal line is the pass mark.

As well as allowing the fixed effect, negative exponential model fitted earlier as M3, SAS also allows a random effects version of the same model, where all three parameters (the starting point, the maximum level and the rate of improvement) are all random rather than fixed variables, so that each can differ between candidates. Details of the program for fitting Model M5 are provided in Additional File 1, and here only a summary of the key results is provided. All of the means, standard deviations and correlations are significantly different from zero with *P *< .001. The starting level has an average of -4.59, with a 95% range from -26.6 to 17.4, which is very similar to that found in M4. However, M5 also assesses the maximum level of attainment, which has a mean of +9.77, with a 95% range from -12.6 to 32.2, meaning that some candidates have a maximum level of achievement which is negative and, hence, substantially below that of the pass mark (zero). There is also variation between candidates in the rate of growth, which has a mean value of -.3178 (the negative value meaning that most candidates approach their maximum level). The 95% range is from -.999 to +.363; the candidates with a value of -.999 approach their maximum level very quickly, whereas the minority of candidates with a positive slope become worse with each attempt. The SAS model also includes correlations between the starting point, maximum level and rate of growth, each of which has its own interpretation. The starting value correlates +.534 with the maximum value, so that those who start at lower values rise to lower values. The starting value also correlates -.228 with the slope, and that correlation requires interpreting with care, since although it means that the slope is lower in those who start at lower values, because slopes in the negative exponential model are negative, a lower (that is, more negative) slope means a greater rate of increase across attempts. Finally, the slope correlates +.357 with the maximum value, and again that must be interpreted with care, and it means that the higher the maximum value, the more slowly it is approached. Interpreting such a set of parameters is not easy and is most clearly seen by estimating the likely curves for candidates across the range of starting abilities. Figure 10 shows typical curves for candidates who are from 30 marks below to 25 marks above the pass mark at their first attempt. The maximum levels are much lower for candidates starting low than for those starting high, but the rate of growth is higher for those starting low (and for those starting high there is barely any growth at all, perhaps not surprisingly since they are already performing extremely well). Note that even though candidates who gain a mark of, for example, 20, at their first attempt will actually not take the examination again, the model is happy to estimate what their marks would have been on repeated attempts, knowing what it knows about other candidates on repeated attempts. Such predictions are in effect extrapolations, albeit extrapolations based on theoretically-driven models, and therefore should be treated with care. They are included here in order to demonstrate what the model is saying about the underlying processes in candidates.

Table 1 shows estimates of the various parameters of Model M5 for Part 1 (which have already been described), as well as for the Part 2 and PACES examinations. Similar effects for Part 1 are found for Part 2 and PACES, although there is less power for detecting correlations in the PACES examination in particular, as fewer candidates make large numbers of attempts. For all three parts of the examination there is variance in the starting level, the maximum level and the rate of improvement in performance, and a correlation of the starting level with the maximum level. The rate of improvement correlates negatively with the starting level in all three parts, although the correlation is significant in only two. The rate of improvement correlates significantly with maximum level in only one of the three parts.

**Table 1:** Estimates of effects in Model 5 for Part 1, Part 2 and PACES.

		Part 1	Part 2	PACES
Starting value (s)	Mean (fixed effect)	-4.60 (.025 ***)	3.14 (.051 ***)	-1.120 (.0474 ***)
	Standard deviation (random effect)	11.2 (.048 ***)	6.63 (.046 ***)	4.98 (.0614 ***)
Maximum (m)	Mean (fixed effect)	9.77 (0.475 ***)	6.33 (.349 ***)	4.42 (.989 ***)
	Standard deviation (random effect)	11.43 (.458 ***)	6.00 (.367 ***)	4.64 (.512 ***)
Rate of growth (b)	Mean (fixed effect)	-.318 (.025 ***)	-.376 (.046 ***)	-.396 (.098 ***)
	Standard deviation (random effect)	.375 (.025 ***)	.869 (.219 ***)	.203 (.148 NS)
Correlation of Starting value with Maximum value		.534 (.029 ***)	.169 (.065 **)	.668 (.082 ***)
Correlation of Starting value with Rate of Growth		-.228 (.044 ***)	-.274 (.117 *)	-.946 (.009 NS)
Correlation of Rate of Growth with Maximum		.357 (.053 ***)	.167 (.153 NS)	-.337 (.787 NS)

Standard errors are indicated in brackets, along with the significance level: *** *P *< .001; l ** *P *< .01; * *P *< .05; NS Not significant. PACES, Practical Assessment of Clinical Examination Skills.

### Model 6: Simultaneous MLwiN modelling of Part 1, Part 2 and PACES

Some candidates start at a low level of performance on the Part 1 examination and only pass it after a number of attempts. An important question, therefore, in a multi-stage examination is how those candidates then perform at Part 2 and PACES. Do they carry forward their underperformance at Part 1, or do they start the next part, as it were, with a clean slate? That question is of particular interest as Part 2 is, in many ways, a more advanced version of Part 1 and, therefore, carryover effects may well be expected between the assessments, whereas PACES is a very different type of examination, assessing mainly practical skills and knowledge, rather than the more theoretical knowledge assessed in Parts 1 and 2, so carryover may not be expected, or at least not expected to such a great extent.

Model-fitting so far has been carried out separately for Part 1, Part 2 and PACES and that has been convenient for expository purposes. However, the candidates who take PACES have previously taken Part 2 and those taking Part 2 have previously taken Part 1, and, therefore, it makes sense to model all three examinations in a single model. In principle that could be done using SAS, fitting models similar to Model 5 but to Part 1, Part 2 and PACES simultaneously, with a random effects model being fitted to the starting value, the rate of growth and the maximum for each of the three exams, and with co-variation between those parameters. Although that should indeed be possible, in practice, after many attempts with SAS, we have been unable to get the program to converge properly, with errors arising from negative eigenvalues. A different approach has therefore been adopted. Model M6 used MLwiN, and concentrated only on the more typical candidates who had taken up to four attempts at each diet.

**Figure 11:**
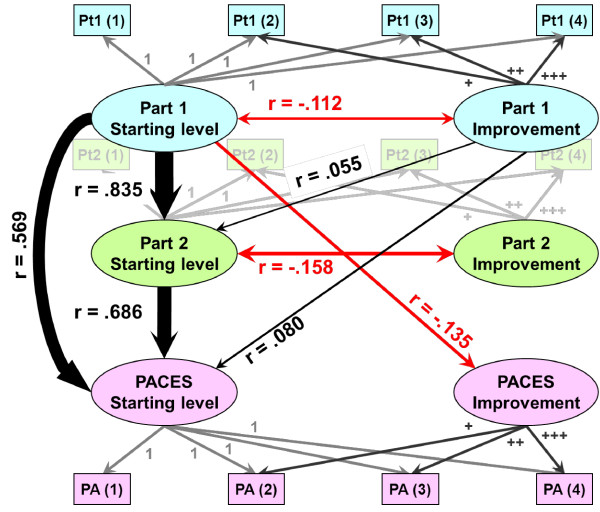
**A summary of model M6, which uses MLwiN to fit nonlinear growth curves simultaneously to the first four attempts at Part 1, Part 2 and PACES (see text for method)**. The raw measures are shown as small rectangles (Pt1(1), for the first attempt at Part 1, and so on), but for clarity are made very faint for Part 2. The six random variables indicating different starting values and rates of improvement at each of the three examinations are shown in the ovals. Correlations between the random variables are shown as double-headed arrows for effects within an examination and single-headed arrows where one examination normally takes place ahead of another examination, and the correlation can be interpreted causally. The strengths of correlations are shown by the thickness of the lines, with positive correlations in black and negative correlations in red. MLwiN, Multilevel Modelling for Windows; PACES, Practical Assessment of Clinical Examination Skills.

The technical details of the analysis of Model M6 are provided in Additional File 1 and here the account will be much more descriptive. The results are presented in Figure 11, which is drawn in the spirit of structural equation models. It will be remembered that MLwiN can only fit linear models and, therefore, the curves of Figure 8 have been linearized using a simple method. As an example, consider just Part 1. A normal linear model, such as that shown in Figure 9, models each result in terms of an overall intercept (performance on the first attempt, or Starting Level as it is called in Figure 11), and a linear function of performance on later attempts (called Improvement in Figure 11), modelled as a multiplier, Beta_1j _times the attempt number, attempts 1, 2, 3, 4, and so on being modelled as the linear series, 0, 1, 2, 3, and so on (and those values are in the variable Attempt0). Attempt0 increases by the same size step at each attempt, whereas Figure 8 shows that the increments decrease in size at each step, so that the overall level of performance tends to an asymptote. For a typical Part 1 candidate, performance on the second, third and fourth attempts is found empirically to be 4.205, 7.028 and 9.407 points higher than on the first attempt. The variable Pt1nonlinearAttempt0, therefore, takes the values 0, 4.205, 7.028 and 9.407 for attempts one to four, being multiplied by a random variable *B*eta_1j_, the mean of which should be about 1 (since 4.025, 7.028 and 9.407 are the means of all the candidates being analyzed here). Some candidates will grow at a greater rate and others at a lesser rate, so that a value of Beta_1j _of, for example, .1, would correspond to a candidate whose performance at the second, third and fourth attempts was only 0.403, 0.703 and 0.941 points higher than at baseline. Differences in the rate of growth are allowed for but candidates only differ in the maximum, asymptotic levels they achieve in relation to a scaling of the entire curve.

Model M6 also carries out the same process for Part 2 and PACES, so that the values for attempts one, two, three and four for Pt2nonlinearAttempt0 and PACESnonlinearAttempt0 are set at 0, 2.666, 4.338 and 5.664, and 0, 2.027, 3.124 and 4.031, respectively. This is shown in Figure 11. The four attempts for Part 1 (labelled Pt1(1), Pt1(2), and so on) are determined by the Part 1 starting level (shown as 1s), and the Part 1 Improvement (symbolised by +, ++ and +++, which actually correspond to values of 4.025, 7.028 and 9.407). The same processes occur for Part 2 and PACES, but to prevent the diagram being too complicated the boxes and arrows for Part 2 are shown very faintly in the background. Model M6 was fitted initially (see Additional File 1 for details), with all co-variances between the six parameters (that is, start and improvement for each of the three examinations) being included in the model. Co-variances which were not significant with *P *< .05 were then dropped from the model until only significant co-variances remained, and these are shown in Figure 11.

For ease of interpretation, co-variances between the parameters are indicated in Figure 11 as correlations and with the width of the line proportional to the size of the correlation. The effects can be broken down into four groups:

1. The largest effects are for the starting levels, a high starting level at Part 2 being predicted strongly by a high starting level at Part 1, and a high starting level at PACES being predicted separately by high starting levels at both Part 1 and Part 2.

2. As in previous models, there are negative correlations, for both Part 1 and Part 2, between the starting level and improvement (that is those who start with lower values improve at a greater rate than those starting at a higher level, as graphed in Figure 10, and as also can be seen in Figure 5). For PACES the correlation between the starting level and improvement was not significant but there was instead a negative correlation of improvement at PACES with the starting level at Part 1.

3. The rates of improvements at the three parts did not show correlations with one another (that is, improving quickly at one examination did not relate to improving quickly at subsequent examinations).

4. There were significant but quite small influences of the rate of improvement at Part 1 on the starting levels at Part 2 and PACES; in other words, whatever the starting level at Part 1, those who improved most quickly subsequently started at a somewhat higher level when they took Part 2 or PACES.

### The impact of centering method

At the suggestion of a reviewer, and in order to assess the impact of having decided not to centre our results, we re-ran two of the analyses using centering around the grand mean (CGM) and centering within clusters (CWC), rather than the RAM (raw measures) analyses reported above. The simple linear model of Figure 9 provided broadly similar estimates for most parameters, with the key exception of the correlation of slope and intercept. With RAM this correlation (the covariance of -.177 in Figure 9) has a non-significant value of -.009. In contrast, with CGM the correlation is a highly significant +.190, and with CWC it is a much larger, and very highly significant +0.730. An analysis of the more complex model presented in Figure 11 and Additional File 1 Figure S2 shows similarly large differences, the correlations between the various slopes and intercepts, which were in the range -.158 to +.080 in the RAM model (see Figure 11), were between -.198 and +.199 (median = -.058) for the CGM model and in the range +.261 to +.872 (median .569) for the CWC model. These large differences can be interpreted following the comments of Enders and Tofighi [23]. CWC emphasizes the relationship between level 1 measures (examination mark and attempt number in this case) after having removed effects due to confounding from level 2. The correlation of 0.730 for the simple linear model is, therefore, asking about the overall relationship; it is asking whether, within candidates, examination marks are higher at later attempts and the (rather obvious) answer is that they are, candidates tending to improve at each resitting. That though is not the primary substantive interest of this study, which concerns not differences in marks between attempts, but the differences in overall performance of candidates, which is summarized in level 2 variables, and in particular their estimated parameters of initial level of performance, rate of growth and asymptote. For such level 2 variables the appropriate analysis uses RAM (and is easier to interpret than CGM, since mean attempt number overall, which is not even an integer, has little obvious meaning), and, at least for the simple linear model of Figure 9, there is no correlation between starting level and rate of improvement.

## Discussion

Examinations in medicine, be they postgraduate or undergraduate, play a key role in ensuring that the technical competence of those passing is at a sufficiently high level to ensure the safe treatment of patients. Implicit in that description is the assumption that the examinations are valid examinations. Validity for postgraduate examinations is currently couched almost entirely in terms of construct validity in its broad sense [27]. Until recently, however, much of the validity of medical examinations has depended on construct validity in the older, narrower sense, in which the items asked about in an examination have a logical and theoretical relationship to medical practice (and essentially, it seems self-evident that, for example, knowledge of the causes and treatment of medical problems such as myocardial infarction, or diabetes, or Fabry's Disease, is more likely to make a better physician than ignorance of such matters). If the knowledge asked about concerns the obscure, recondite, 'fascinomas' once beloved of some examiners, then construct validity in the narrow sense may not necessarily be the case. Excluding that type of question, it is hard to make an argument, beyond mere hand-waving and a few splutters about 'only exam knowledge', that those who have a greater knowledge of medical conditions are no more likely to be better doctors than those who do not have such knowledge. With well-constructed, properly blue-printed examinations (part of the broad sense of construct validity), it seems more likely to be true for physicians that knowledge is better than ignorance. In the case of the MRCP(UK), educators and, particularly, future patients might reflect on whether they would genuinely be indifferent as to whether their physicians did not know about, for example, aseptic meningitis in infectious mononucleosis, bone marrow changes in chronic anaemia of infection, or the electrophysiology of Wolff-Parkinson-White syndrome [28]. When it is asked whether examinations are 'valid', the question is often referring only to predictive validity, which would require a demonstration that those who do better on postgraduate examinations subsequently perform better as doctors on concrete outcomes in daily medical care (or more particularly, that those who do less well show less good care). At present there are almost no studies which have looked at predictive validity (and matters have not changed much since the review of Hutchinson *et al. *[29]), although at present we are carrying out a number of studies on the predictive validity of MRCP(UK) in relation to future professional behaviour and clinical practice, and hope to publish in the future. The present study is not, however, looking at predictive validity for future medical care, but is concerned instead with the examination itself and its correlates. There is however an implicit assumption that the examination is valid, particularly in the sense of construct validity.

If examinations are high-stakes, then natural justice requires that if examinations are difficult, and a doctor cannot continue in their chosen specialty without having passed those examinations, that the examinations be fair, valid and reliable (and see Mehrens and Popham [30] for a good overview of the legal issues involved). On the particular issue of resit assessments in high-stakes assessments, the review cites a court case on teacher assessments in the US State of Georgia, in which the judgment stated that,

'[an] irrebuttable lifetime presumption of unfitness after failure to pass six [assessments] was arbitrary and capricious because no further education, training, experience, maturity or higher degree would enable such persons to become certified ...' [30] [p.270].

It is also worth noting that the phrase 'arbitrary and capricious' also forms a part of some university regulations on examination assessment (see for example, http://www.umuc.edu/policies/academicpolicies/aa13080.cfm). 'Arbitrary' and 'capricious' have been defined in a legal context as,

'A rule is arbitrary if it is not supported by logic or the necessary facts; a rule is capricious if it is adopted without thought or reason or is irrational' http://definitions.uslegal.com/a/arbitrary-and-capricious/.

Within medical education, and particularly in the context of setting standards or pass marks, it is a commonplace to find phrases such as that of Case and Swanson [31], who say, 'Setting standards will always be arbitrary but need not be capricious' (p.111). Certainly at first sight there does seem to be some arbitrariness whenever a continuum of marks is divided at some cut point to distinguish those who pass and those who fail. However, in the sense of being, 'not supported by logic or the necessary facts', there is surely a strong argument that well designed pass marks, perhaps based on clear criterion referencing, or on the Angoff, Edel or Hofstee methods, or on statistical equating, are not arbitrary, since they are grounded in principle, method, evidence and logic, with a carefully articulated measurement model. There might be those who would argue that a pass mark is too strict or too lax, but that is a separate issue from the rational basis by which the pass mark itself has been set.

Part of the process of fairness and natural justice is that if a candidate fails an examination at one attempt, particularly if they feel they were unlucky in an earlier attempt, perhaps because of a particular choice of questions they had been asked (that is, content specificity/case specificity [32-34]), then they should be allowed to resit the examination. At that point the difficult question arises of how many times a candidate should be allowed to resit. In the late 1990s, the MRCP(UK) decided, given the then available evidence, that it could see no reasonable academic argument to prevent candidates from taking an examination as many times as they wished, particularly given that the standards of its examinations were high and the examinations were reliable, particularly for Part 1 [35]. As an extreme example, one candidate in our database subsequently had a total of 35 attempts across the three examinations before eventually gaining the MRCP(UK). Since the candidate had eventually met our standards at each examination there is an argument that it would not have been justified to prevent their progress arbitrarily at an earlier stage.

Although some of the MRCP candidates taking assessments ten or even twenty times may seem extreme in their numbers of attempts, occasional accounts exist of candidates who pass examinations after a very much greater number of attempts, particularly with computer-based assessments. A report on the BBC website http://news.bbc.co.uk/1/hi/8347164.stm described the case of Mrs Cha Sa-Soon, a 68-year-old woman who had passed the theory part of the driving test of South Korea at her 950^th ^attempt. The multiple choice examination has a pass mark of 60% and consists of 40 questions, according to the *New York Times *http://www.nytimes.com/2010/09/04/world/asia/04driver.html. When an examination can be taken every day, as can the South Korean driving test, it might seem dubious that a genuine increase in ability has continued to occur until the 950^th ^attempt and it may be thought that chance had begun to play a substantial role. That being said, if the examination were best-of-four, giving a 25% chance of success on any question, and if there were 40 questions, the probability of attaining 60% correct by responding at random would only be about 1 in 1.7 million. The likelihood of success by chance alone by the 950^th ^attempt is quite low, implying that Mrs Cha had not passed entirely due to luck (and the *New York Times *did say that, 'her scores steadily crept up'). (It should be noted that for examinations such as driving tests there is typically a finite pool of questions, which are themselves sometimes published in their entirety, so that rote learning of the answers is in principle possible).

Calculations for the probability of correctly answering sufficient questions to pass in the 200 best-of-five questions at MRCP(UK) Part 1 suggest it would be extremely unlikely that a candidate could pass merely due to luck alone. At this point it is perhaps worth quoting from the paper by Pell *et al. *[[4], p.249], who say:

'The question has often been put to the authors, 'Are not OSCEs [and other assessments] rather like the driving test, candidates are required to reach a certain level of competence, and their route is of little consequence?' In other words, this argument implies that students should be allowed as many resits as necessary until they reach the appropriate level of competence'.

However, Pell *et al. *resist the obvious conclusion and say they, 'are strongly of the opinion that resits should be constructed to take at least some account of the additional time and support that resit students have been afforded'. How to do that is not straightforward and will be considered in detail elsewhere.

The present study provides a substantial empirical contribution to the evidence base on repeated testing. By means of multilevel modelling of the extensive records of the MRCP(UK), it manages to provide numerical estimates of the extent to which the true ability of candidates improves at repeated attempts at an examination and, hence, the extent to which luck rather than ability begins to play a role. In relation to the central statistical question of the role of luck and genuine improvement, it is clear that on average there is a genuine improvement over many attempts at examinations. It should also be remembered that luck might help an individual candidate pass on a particular attempt but on average it should not increase the overall mark of candidates; that requires a genuine increase in knowledge.

For the Part 1 examination, for which the range of abilities is necessarily much wider, candidates are, on average, still improving at their tenth attempt at the examination. More sophisticated modelling suggests that there is a maximum level of achievement for each candidate, that the maximum level differs between candidates and is sometimes below the pass mark, making eventual success highly unlikely, and that the maximum level correlates strongly with the mark attained at a first attempt at the examination (see Figure 10 for an illustration). Furthermore, the mark attained at a first attempt at the Part 2 and PACES examinations, the taking of which is contingent upon success in the Part 1 examination, depends strongly upon the mark at the first attempt at Part 1, but not on the improvement that subsequently occurs until Part 1 is eventually passed.

In the UK the question of whether candidates in postgraduate examinations should be limited in their number of attempts at an examination has historically been at the discretion of individual examining bodies. The same is also true of undergraduate examinations, where it is generally the case at present that only one or perhaps two attempts at finals or other examinations are allowed (although historically it has not always been so). The rationale for whatever regulations apply is often far from clear and the impression is that whatever limit there is has little formal basis in theory. The primary theoretical concern has to be with the role of 'luck', a difficult term to use, which is partly random variation due to the candidate (perhaps feeling ill on the day, or whatever), partly random variation due to the examiners (who also may feel jaundiced on the day), or the content of the questions (content/case specificity), or can be a deeper process that can simply be regarded as 'chance', 'random variation', 'measurement error', or whatever. The concept of 'luck' is subtle, but consider two candidates, one of whom A, knows about condition P but not Q, and the other B, who knows about condition Q but not P, so both know about half of the expected knowledge. Condition P is asked about, and so A passes but B fails, but on the next occasion the examination asks about Q, and so at the resit B passes. A finite examination cannot ask about all conditions, and so A was indeed lucky (and A's future patients with condition Q could also be regarded as unlucky). B was also lucky that Q eventually came up. Good examinations try to reduce all such factors by blue-printing, ensuring that the examination contains a large, representative number of questions across the entire syllabus, but they can never be entirely eliminated.

The role of purely 'chance' factors is most easily seen in an outcome which depends entirely on chance, as in dice games, where one has to throw a single die to get a six. There is a one in six chance of throwing a six on the first attempt, but with every additional throw the probability of eventually throwing a six increases. However, that increased probability increases with every additional throw. Likewise, the probability of passing an examination due to chance components (and that includes having 'got lucky' due to not feeling ill, examiners feeling beneficent, and cases/questions with which one happens to be experienced) increases with every additional attempt. There is no discrete change in the probability at the seventh (or indeed any other) specific attempt. More problematic is that the probability of passing due to luck begins to rise even at the second attempt (when many candidates do indeed pass examinations which they have failed at their first attempt). Any proper solution to the problem of resits has, therefore, to consider the difficult problem of whether there is a need to set a gradually increasing pass mark for each attempt at an examination, so that a mark which would pass a candidate at their first attempt may result in a failure at a later attempt, even be it their second attempt (when luck has already begun to benefit the candidate).

The central question underpinning any policy on numbers of resits has to be whether a limit is capricious, that is, 'if it is ... irrational', and that is where the difficult problem lies for medical examiners. The fundamental problem in understanding resit examinations is that at any attempt the mark of a candidate is a combination of their true ability and a random, chance process. With each and every repeated attempt at an examination, a candidate capitalizes on those random, chance processes, so that as the number of attempts increases, the probability of benefitting from chance increases with each and every attempt. It is not, therefore, rational or logical to implement a process which implicitly assumes that chance plays no increasing role on attempts one to N, but it does play a role from attempt N+1 onwards, so that N is the limit on attempts allowed. The laws of probability are not compatible with such an approach and, therefore, the process cannot be rational. In socio-political terms, the proposed limit of N appears to find its origins partly as an administrative convenience but mainly as an attempt to provide reassurance. However, that reassurance is surely false and without substance, not only because it does not correctly take chance into account, but because empirically it is the case that most candidates who pass at resits do so at the second or third attempt, when chance will almost certainly have benefitted a proportion of them, and the limit of N does nothing to impede those individuals. Candidates currently passing at, for instance, the seventh or higher attempt are a small minority of those passing at resits.

While there is no rational basis for having a fixed limit to the number of attempts, neither is the converse rational, of allowing an unlimited number of attempts, since chance continues to benefit resit candidates and that will not reassure the public. There is, though, a third way, which is perhaps the only possible rational solution, which is to set a pass mark that itself is dependent on the number of attempts an individual candidate has made. Indeed, an argument could be made, from a Bayesian perspective, that the pass mark for an individual candidate should be dependent on the marks they have obtained at all previous attempts at an examination, a candidate who has previously failed badly having to do better at the Nth attempt than one who only had bare fails on previous attempts. Although far from straightforward to implement, given that any other process could be argued to be capricious, then it is the only solution which can claim to be rational, to avoid the claim of being capricious, and also to be seen to be protecting and reassuring patients.

## Conclusions

Candidates continue to show evidence of true improvement in performance up to at least the tenth attempt at MRCP(UK) Part 1, although there are individual differences in the starting level, the rate of improvement and the maximum level that can be achieved. Such findings provide little support for arguments that candidates should only be allowed a fixed number of attempts at an examination. However unlimited numbers of attempts are also difficult to justify because of the inevitable and ever increasing role that luck must play with increasing numbers of resits, so that the issue of multiple attempts might be better addressed by tackling the difficult question of how a pass mark should increase with each attempt at an examination.

## Abbreviations

AR: auto-regressive; ARIMA: auto-regressive integrated moving average; BBC: British Broadcasting Corporation; BOF: Best-of-Five (examination); CGM: centering to grand mean; CWC: centering within clusters; DPM: Diploma in Pharmaceutical Medicine; DRCOG: Diploma of the Royal College of Obstetrics and Gynaecology; FCEM: Fellowship of the College of Emergency Medicine; FRCA: Fellowship of the Royal College of Anaesthetists; FRCR: Fellowship of the Royal College of Radiologists; FRCS: Fellowship of the Royal College of Surgeons; GMC: General Medical Council; MA: moving average; MCEM: Membership of the College of Emergency Medicine; MFOM: Membership of the Faculty of Occupational Medicine; MLM: multi-level modelling; MLwiN: Multilevel Modelling for Windows; MRCGP: Membership of the Royal College of General Practitioners; MRCOG: Membership of the Royal College of Obstetricians and Gynaecologists; MRCPath: Membership of the Royal College of Pathologists; MRCP(UK): Membership of the Royal Colleges of Physicians of the United Kingdom; MRCPCH: Membership of the Royal College of Paediatrics and Child Health; MRCPsych: Membership of the Royal College of Psychiatrists; MRCS: Membership of the Royal College of Surgeons; MTF: multiple true-false (examination); PACES: Practical Assessment of Clinical Examination Skills; PLAB: Professional and Linguistic Assessments Board (of the GMC); RAM: raw measures (analysis with no centering); SAS: Statistical Analysis System; SPSS: Statistical Package for the Social Sciences; UK: United Kingdom; US: United States; USMLE: United States Medical Licensing Examination.

## Competing interests

ICM is Educational Advisor to the MRCP(UK) and KL is on an Impact Studentship, part-funded by MRCP(UK).

## Authors' contributions

The original idea for the study was ICM's, and organization of the datasets and preliminary analysis of the data was carried out by KL and ICM jointly. Multilevel modelling using MLwiN was carried out jointly by KL and ICM, and SAS modelling was carried out by ICM. The first draft of the paper was written by ICM. Both authors contributed to the final version of the manuscript and the revisions, and both authors read and approved the final manuscript.

## Pre-publication history

The pre-publication history for this paper can be accessed here:

http://www.biomedcentral.com/1741-7015/10/60/prepub

## Supplementary Material

Additional file 1 **Details of fitting of models using MLwiN and SAS**. This file contains technical details on the fitting of the MLwiN and SAS models. Click here for file
