# Supplementary material for: Resitting a high-stakes postgraduate medical examination on multiple occasions: nonlinear multilevel modelling of performance in the MRCP(UK) examinations
Source: BMC Med. 2012 Jun 14;10:60. doi: 10.1186/1741-7015-10-60 (PMC3394208; doi:10.1186/1741-7015-10-60)
Supplement: Additional file 1 — Details of fitting of models using MLwiN and SAS. This file contains technical details on the fitting of the MLwiN and SAS models. [file 1741-7015-10-60-S1.PDF]

Details of fitting of models using MLwiN and SAS

Model 1. Model 1 is a simple multilevel model of data from MRCP(UK) Part1, and is shown in annotated form as figure 6 of the main paper. The two levels are measurements (level 1), and candidates (level 2), and the effects of attempt are modelled only as a random effect of starting level and a fixed effect of rate of growth (parameterised as *Pt1Attempt0*, for which the first attempt is 0, the second attempt is 1, and the *n*th attempt is *n-1*. For other information see the main paper. The model has 4 parameters and a Deviance (-2 x Log.Likelihood of 513604.899). In practical terms, the data for *MLwiN* were imported as an *SPSS* .sav file, with one record for each attempt, and candidate numbers being coded so that *MLwiN* knew about repeated attempts by individual candidates.

Model 2. Model 2 is similar to Model 1 except that instead of growth being modelled as a single linear function of attempt number, there are 19 dummy variables, one for each attempt up to the twentieth, with an estimate of each so that curvilinearity can be visually assessed. The fitted model is shown in Additional File, Figure S1, and has a Deviance of 510551.394, based on 22 parameters, making a difference from model M1 of 3053.5, which is a chi-square statistic with 18 df, and very highly significant. The estimates of the dummies, along with their 95% confidence intervals are shown in figure 7 of the main paper.

Model 3. Model 3 is similar to Models 1 and 2, except that instead of a purely linear growth on attempt, or a piece-wise fitting of attempt number via dummies, the curve is fitted directly by means of a non-linear model which is a negative exponential. Details are not provided here since the model is a subset of the more complex models described later.

The SAS code for fitting the model is shown below. Note that: *s* = *Start*, *m* = Max, *b* = Slope, and variables ending in a *j* are random variants of the fixed variables of the same name. Variances are fitted as standard deviations (beginning *SD*), and hence are squared to generate variances. Some variables have bounds, with Slope having to be less than zero, and the SD having to be greater than zero. Method=FIRO is not essential, but sometimes allows the model to converge when it would not do so otherwise. The only data variables are *Pt1Mark*, *Pt1Attempt* and *CandNo*, each row of the data consisting of a single candidate on a single attempt.

```
PROC NLMIXED method=firo ;
     Startj=Start+Startj;
     yhat=Max - (Max-Startj)*exp((Pt1attempt-1)*Slope);
     MODEL Pt1Mark ~ NORMAL (yhat,SDwithin*SDwithin);
     RANDOM Startj ~ NORMAL ( 0],[SDstart*SDstart])
          SUBJECT = CANDNO ;
     PARMS  SDwithin=5  Start = -5 Max = 10
          Slope=-.25 SDstart=10 ;
```

```
     BOUNDS Slope < 0, SDstart > 0;
RUN;
```

Model 4. The fitted version of Model M4 is shown as figure 9 in the main paper. No further comment is needed here.

Model 5. Random effects negative exponential model in SAS. The SAS code for fitting the model is shown below. Note that: *s* = *Start*, *m* = Max, *b* = Slope, and variables ending in a *j* are random variants of the fixed variables of the same name. Variables beginning with *R* are correlations, and variances are fitted as standard deviations (beginning *SD*), and hence are squared on the diagonal of the covariance matrix. Some variables have bounds, with Slope having to be less than zero, and the SDs having to be greater than zero. Method=FIRO is not essential, but sometimes allows the model to converge when it would not do so otherwise. The only data variables are *Pt1Mark, Pt1Attempt* and *CandNo*, each row of the data consisting of a single candidate on a single attempt.

```
PROC NLMIXED method=firo ;
     Startj=Start+Startj;
     Maxj=Max+Maxj;
     Slopej=Slope +Slopej;
     yhat=Maxj -(Maxj-Startj)*exp((Pt1attempt-1)*Slopej);
     MODEL Pt1Mark ~ NORMAL (yhat,SDwithin*SDwithin);
     RANDOM Startj Maxj Slopej ~ NORMAL ([0,0,0] ,
          [SDstart*SDstart,
          RstartMax*SDstart*SDmax, SDmax*SDmax,
     RstartSlope*SDstart*SDslope,
     RmaxSlope*SDmax*SDslope, SDslope*SDslope]
          ) SUBJECT = CANDNO ;
     PARMS SDwithin=5  Start = -5 Max = 10 Slope=-.25
          SDstart=10  SDmax=10  SDslope=.4 RstartMax =0
RstartSlope=0 RmaxSlope=0 ;
     BOUNDS Slope < 0, SDstart > 0, SDmax > 0,
          SDslope > 0 ;
RUN;
```

Model M6. Model M6 is fitted in *MLwiN* and uses data from all candidates on attempts 1 to 4 of any part of the exam. It takes into account the performance of candidates at Part 2 in relation to performance at Part 1, and at PACES in relation to Part 1 and Part 2. Additional File, Figure S2 shows the Equations Window of *MLwiN* and gives both the equations that are fitted, and the estimates of the parameters. Two sets of random factors are used in the model. The first set, *Pt1constant*, *Pt2constant* and *PACESconstant* correspond to the intercept, and hence to the *Starting Level* at each part (as it is called in Figure 11 of the main paper). *Pt1NonlinAttempt*, *Pt2NonlinAttempt* and *PACESnonlinAttempt* correspond to the curvilinear functions fitted previously in model M5, and for attempts 1,2, 3 and 4 have values of 0, 4.205, 7.028 and 9.407 for Part 1, 0, 2.666, 4.338 and 5.664 for Part 2, and 0, 2.027, 3.124 and 4.031 for PACES. They correspond to an average trajectory for a candidate, and hence a loading of 1 indicates that the candidate is progressing at a typical rate. The *Nonlin* components are what in Figure 11 of the main paper are called *Part 1 Improvement*, etc.. The

model was fitted with all elements of the level 2 covariance matrix, and then off-diagonal elements were dropped in turn, with the least significant first, until all of the elements were significant. Correlations (not shown) were then calculated using the Tables command in *MLwiN* and used to plot Figure 11 in the main paper. In practical terms, the data for *MLwiN* were imported as an *SPSS* .sav file, with one record for each attempt at any part of the exam, and examination part being indicated by whether the Constants for each Part were zero or one (and if they were one they were included in the model, and zero they were ignored for that part). Candidate numbers were also included so that *MLwiN* knew about repeated attempts by individual candidates across the different parts.

Additional File 1, Figure S1

$$\text{Pt1Mark}_{ij} \sim \text{N}(XB, \ \Omega)$$

$$\begin{aligned}\text{Pt1Mark}_{ij} = \ & \beta_{0ij}\text{Constant} + 4.125(0.057)\text{dummy2}_{ij} + 2.917(0.074)\text{dummy3}_{ij} + \\ & 2.078(0.097)\text{dummy4}_{ij} + 1.844(0.125)\text{dummy5}_{ij} + 1.256(0.161)\text{dummy6}_{ij} + \\ & 1.067(0.202)\text{dummy7}_{ij} + 0.921(0.251)\text{dummy8}_{ij} + 0.733(0.310)\text{dummy9}_{ij} + \\ & 0.930(0.381)\text{dummy10}_{ij} + 0.700(0.462)\text{dummy11}_{ij} + 0.247(0.544)\text{dummy12}_{ij} + \\ & 0.091(0.642)\text{dummy13}_{ij} + 1.851(0.764)\text{dummy14}_{ij} + -0.475(0.911)\text{dummy15}_{ij} + \\ & 0.580(1.087)\text{dummy16}_{ij} + -0.326(1.347)\text{dummy17}_{ij} + 0.541(1.581)\text{dummy18}_{ij} + \\ & 0.589(1.909)\text{dummy19}_{ij} + 1.955(2.283)\text{dummy20}_{ij}\end{aligned}$$

$$\beta_{0ij} = -4.681(0.062) + u_{0j} + e_{0ij}$$

$$\left[u_{0j}\right] \sim \text{N}(0, \ \Omega_u) \ : \ \Omega_u = \left[121.544(1.000)\right]$$

$$\left[e_{0ij}\right] \sim \text{N}(0, \ \Omega_e) \ : \ \Omega_e = \left[23.646(0.188)\right]$$

*-2*loglikelihood(IGLS Deviance)* = 510551.394(70856 of 70856 cases in use)

Additional File 2, Figure S2

$$\text{Mark}_{ij} \sim \text{N}(XB, \Omega)$$

$$\text{Mark}_{ij} = \beta_{0ij}\text{Pt1Constant}_{ij} + \beta_{1ij}\text{Pt2Constant}_{ij} + \beta_{2ij}\text{PACESconstant}_{ij} + \beta_{3j}\text{Pt1NonlinAttempt0}_{ij} + \beta_{4j}\text{Pt2NonlinAttempt0}_{ij} + \beta_{5j}\text{PACESnonlinAttempt0}_{ij}$$

$$\beta_{0ij} = -4.317(0.059) + u_{0j} + e_{0ij}$$

$$\beta_{1ij} = 0.862(0.048) + u_{1j} + e_{1ij}$$

$$\beta_{2ij} = -2.446(0.047) + u_{2j} + e_{2ij}$$

$$\beta_{3j} = 1.023(0.009) + u_{3j}$$

$$\beta_{4j} = 1.004(0.016) + u_{4j}$$

$$\beta_{5j} = 1.005(0.021) + u_{5j}$$

$$\begin{bmatrix} u_{0j} \\ u_{1j} \\ u_{2j} \\ u_{3j} \\ u_{4j} \\ u_{5j} \end{bmatrix} \sim \text{N}(0, \Omega_u) : \Omega_u = \begin{bmatrix} 123.168(1.046) & & & & & \\ 69.322(0.675) & 55.981(0.643) & & & & \\ 33.987(0.636) & 27.617(0.408) & 28.956(0.450) & & & \\ -0.675(0.112) & 0.222(0.082) & 0.233(0.087) & 0.293(0.016) & & \\ -0.479(0.179) & -0.656(0.130) & 0 & 0 & 0.307(0.038) & \\ -0.591(0.220) & 0 & 0 & 0 & 0 & 0.156(0.049) \end{bmatrix}$$

$$\begin{bmatrix} e_{0ij} \\ e_{1ij} \\ e_{2ij} \end{bmatrix} \sim \text{N}(0, \Omega_e) : \Omega_e = \begin{bmatrix} 19.479(0.231) & & \\ 0 & 13.742(0.237) & \\ 0 & 0 & 23.416(0.271) \end{bmatrix}$$
